# Impact of Elovl3 Suppression on Sleep Deprivation-Induced Hepatic Steatosis and Glucose Intolerance in Mice

**DOI:** 10.7759/cureus.107228

**Published:** 2026-04-17

**Authors:** Fumika Shigiyama, Sayaka Tanaka, Chiaki Okayama, Naoki Kumashiro

**Affiliations:** 1 Diabetology and Endocrinology, Kanazawa Medical University, Ishikawa, JPN

**Keywords:** elovl3, glucose intolerance, hepatic steatosis, insulin resistance (ir), japanese geriatrics, sleep deprivation

## Abstract

Background

Sleep plays a key role in glucose homeostasis and is an essential component of metabolic health. Sleep deprivation can induce hepatic steatosis and insulin resistance in mice, and these effects are thought to involve disruption of circadian metabolic regulation and neuroendocrine pathways. Sleep deprivation has also been associated with increased hepatic expression of elongation of very long chain fatty acids-like 3 (*Elovl3*). Because *Elovl3 *is involved in very-long-chain fatty acid synthesis and exhibits circadian regulation in the liver, we hypothesized that hepatic *Elovl3 *upregulation may contribute to sleep deprivation-induced lipid accumulation. This study aimed to investigate whether downregulation of hepatic *Elovl3* expression influences hepatic lipid accumulation and glucose metabolism under sleep deprivation conditions.

Methods

Eleven-week-old male C57BL/6J mice were fed a high-fat diet and high-sucrose water for two weeks, followed by a single 6-hour period of sleep deprivation. To suppress hepatic *Elovl3* expression, *Elovl3*-specific small interfering RNA (siRNA) was administered via tail vein injection (*Elovl3* siRNA group), whereas control mice received non-coding siRNA (negative siRNA group). Hepatic triglyceride content, gene expression, gluconeogenesis (pyruvate challenge test), insulin sensitivity (insulin tolerance test), and glucose tolerance (intraperitoneal glucose tolerance test (ipGTT)) were evaluated. The primary outcome of this study was hepatic triglyceride accumulation after acute sleep deprivation. Secondary outcomes included glucose tolerance, gluconeogenesis, and insulin sensitivity.

Results

A single 6-hour sleep deprivation increased hepatic *Elovl3* mRNA expression by 2.7-fold; this increase was completely suppressed 48 hours after administration of *Elovl3* siRNA. Hepatic triglyceride content was significantly lower in the *Elovl3* siRNA group than in the negative siRNA group after sleep deprivation (3.1 ± 1.5 vs 10.4 ± 4.2 mg/g tissue, respectively), corresponding to an approximate 70.4% reduction, under fasting and movement-restriction conditions following sleep deprivation. Hepatic gluconeogenesis did not differ between groups, whereas blood glucose levels at 30 minutes during ipGTT were significantly lower in the *Elovl3* siRNA group.

Conclusions

Suppression of hepatic *Elovl3* expression attenuated sleep deprivation-induced hepatic steatosis in mice. However, although the blood glucose level at 30 minutes during ipGTT was significantly lower in the Elovl3 siRNA group, no significant differences were observed in glucose area under the curve during ipGTT, pyruvate challenge test, or insulin tolerance test, suggesting that additional interventions may be required to improve glucose intolerance associated with sleep deprivation. Hepatic *Elovl3* may represent a potential therapeutic target for sleep deprivation-related metabolic dysfunction.

## Introduction

Over the past few decades, sleep disorders have gained attention as a major public health issue [1]. More than 16% of the global population is estimated to have insomnia [2]. Sleep disorders are becoming increasingly prevalent with aging due to age-related changes in circadian rhythm regulation, alterations in sleep architecture, and the increasing burden of comorbid medical conditions [3,4]. Such alterations in sleep patterns, including insomnia and irregular sleep, have been associated with cardiometabolic diseases and other chronic conditions across diverse populations [5,6]. Importantly, sleep disorders have been reported to be independently associated with an increased risk of both type 2 diabetes mellitus (T2DM) and metabolic dysfunction-associated steatotic liver disease, even after adjusting for conventional lifestyle risk factors [7-9]. Therefore, sleep disorders may represent a potential target in prevention and treatment strategies for age-related metabolic diseases.

Several clinical and experimental studies have addressed the association between sleep disorders and glucose intolerance and insulin resistance [10,11]. In rodent models, sleep disorders have been associated with increased food intake [12,13] and reduced whole-body energy expenditure [14]. However, the underlying mechanisms and therapeutic targets for treating sleep deprivation-induced glucose intolerance remain unclear. The pathogenesis of T2DM is associated with peripheral hepatic and muscle insulin resistance due to ectopic lipid accumulation in the liver and skeletal muscle. The liver plays a central role in metabolic homeostasis by regulating gluconeogenesis, glycogen storage, de novo lipogenesis, fatty acid oxidation, and the distribution of circulating nutrients according to feeding-fasting and circadian states. In mammals, the liver is one of the biological circadian clock systems that plays an important physiological role in energy metabolism and nutrient diurnal processing [15,16]. In addition, the liver is a major peripheral clock organ that coordinates daily oscillations in glucose and lipid metabolism [15-18]. Sleep deprivation may therefore promote hepatic steatosis through both circadian dysregulation and neuroendocrine perturbations, including altered autonomic and hormonal signaling [17,19]. Our previous study revealed that a single 6-hour sleep deprivation caused hepatic steatosis and insulin resistance in C57BL/6J mice as an acute model, and a significant increase in hepatic elongation of very long chain fatty acids-like 3 (*Elovl3*­­) gene expression [20]. *Elovl3* is a member of the *elovl* gene family, which encodes an enzyme implicated in the synthesis of C20-C24 saturated and monounsaturated very long-chain fatty acids [21,22].

Furthermore, according to a previous transcriptome analysis, the steady-state levels of *Elovl3* mRNA displayed a robust circadian profile in the liver [20]. Therefore, we hypothesized that by cancelling *Elovl3* expression in the liver, hepatic lipogenesis would be suppressed, which could contribute to the improvement of insulin resistance. Thus, this study aimed to investigate the effects of downregulation of hepatic *Elovl3* expression on hepatic lipid accumulation and glucose metabolism during sleep deprivation. From this perspective, by using *Elovl3-*specific siRNA, we evaluated the effect of cancelling the increase in hepatic expression of *Elovl3* following a single 6-hour sleep deprivation using a gentle handling method as previously described [20]. The primary outcome of this study was hepatic triglyceride accumulation after acute sleep deprivation. Secondary outcomes included glucose tolerance, gluconeogenic response, and insulin sensitivity as assessed by the intraperitoneal glucose tolerance test, pyruvate challenge test, and insulin tolerance test, respectively.

## Materials and methods

Animals

All procedures and protocols used in this study were approved by the Institutional Animal Care and Use Committee of Kanazawa Medical University School of Medicine. Eight- to nine-week-old male C57BL/6J mice were obtained from CLEA Japan Inc. (Tokyo, Japan). They were individually housed in plastic cages under a 12 h light-12 h dark cycle (light on at 08:00) at a controlled ambient temperature (24±1 °C) and were allowed access to food and water *ad libitum*. During the 14-day habituation period, mice were fed a high-fat diet (D12451, Research Diets, Inc., New Brunswick, USA) and 5% sucrose water to induce a prediabetic state, mimicking conditions in which the metabolic effects of sleep deprivation are often exacerbated. All experiments were performed until the mice reached 13 or 14 weeks of age.

Small interfering RNA (siRNA)

Stable *Elovl3* and negative siRNAs obtained from Sigma (St. Louis, USA) were combined with the Invivofectamine™ 3.0 reagent (Invitrogen, Thermo Scientific, USA), according to the manufacturer’s instructions. These complexes (0.5 mg/kg-BW) were injected into C57BL/6J mice through the tail vein 48 h before each experiment, a regimen previously optimized to achieve maximal and sustained hepatic *Elovl3* knockdown at the time of sleep deprivation based on preliminary experiments.

Sleep deprivation protocol

Sleep deprivation was induced as previously described [20], a model chosen for its acute induction of hepatic steatosis and *Elovl3* upregulation in mice. In brief, to avoid any additional movement and stress on the day of the experiment, the mice were immobilized in individual small plastic cages (Natsume Seisakusho Co., Tokyo, Japan) for 6 hours (from 08:00 to 14:00), and their physical activity was limited to a minimum. Mice were continuously monitored visually for signs of sleep, and when they began to close their eyes and recline, the plastic cages were handled by gentle touching. The mice had been fasted since 20:00 the previous night. Tissue and blood sampling, intraperitoneal glucose tolerance, pyruvate challenge, and insulin tolerance tests were performed immediately after the 6-hour sleep deprivation period.

Tissue sampling

At the end of the sleep deprivation period (no recovery), mice were euthanized. Tissues were immediately dissected, flash-frozen in liquid nitrogen, enclosed in aluminum foil, and preserved at −80 °C.

Laboratory tests

Blood glucose concentration was measured using a portable glucose meter (Accu-Chek Aviva Nano; Roche, Basel, Switzerland), consistent with our previous murine metabolic study [20]. Although this type of device has been widely used in murine metabolic experiments [23,24], we acknowledge that the present study did not include an independent device-validation analysis against a laboratory reference method.

Intraperitoneal glucose tolerance, pyruvate challenge, and insulin tolerance tests

At the end of the sleep deprivation period, intraperitoneal glucose tolerance, pyruvate challenge, and insulin tolerance tests were performed as described previously [20,23,24]. Briefly, mice were intraperitoneally injected with 20% glucose (2.0 g/kg-BW) for intraperitoneal glucose tolerance tests (ipGTT), with pyruvate (1.5 g/kg-BW) for the pyruvate challenge test, or with insulin (0.75 U/kg-BW) for the insulin tolerance test. Blood samples were collected from the tail vein at the indicated time, and plasma glucose levels were measured using a portable glucose meter.

Hepatic triglyceride assay

Hepatic triglyceride content was measured using a triglyceride assay kit (Triglyceride E-test WAKO, Tokyo, Japan). Briefly, approximately 50 mg of liver tissue was homogenized in chloroform: methanol (2:1), and lipids were extracted by shaking for 3-4 hours at room temperature (24±4 °C). After the addition of 100 mM sulfuric acid, the samples were vortexed and centrifuged. The organic phase was collected and used for the triglyceride content analysis.

Real-time polymerase chain reaction (RT-PCR)

Total RNA was extracted from flash-frozen liver samples (approximately 20 mg) using the RNeasy mini kit (Qiagen, Tokyo, Japan) and transcribed into complementary DNA (cDNA) using the QuantiTect Reverse Transcription Kit (Qiagen, Hilden, Germany). Transcripts were quantified using real-time PCR on an Applied Biosystems 7500 Fast Real-Time PCR System (Thermo Fisher Diagnostics, Tokyo, Japan) with Fast SYBR Green Master Mix (Thermo Fisher Diagnostics). The expression level of each gene of interest was normalized for the efficiency of amplification, with TATA box-binding protein mRNA as the invariant control, using a standard curve.

Statistical analysis

Data are presented as mean ± standard deviation unless otherwise specified. Statistical significance was set at p < 0.05. Differences among groups were assessed using two-way analysis of variance followed by the Tukey post hoc test for ipGTT and pyruvate challenge tests, and by the unpaired t-test for the other experiments. Statistical analyses were performed using Prism 7 (GraphPad Software, San Diego, USA).

## Results

**Figure 1:**
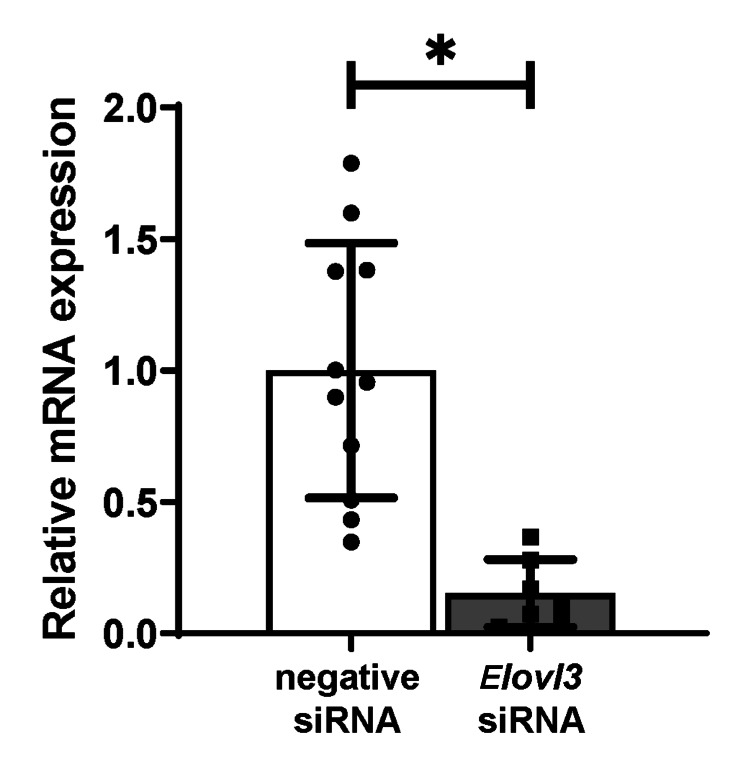
Relative mRNA expression of Elovl3. Hepatic mRNA expression was evaluated after a single 6-hour sleep deprivation. Forty-eight hours before sleep deprivation, *Elovl3* siRNA or negative siRNA was injected. Relative mRNA expression was significantly lower in the *Elovl3* siRNA group (n=7) than in the negative siRNA group (n=11). The average of the negative siRNA group was set as 1. Data values correspond to mean values, and error bars represent the standard deviation of the means. Differences among groups were assessed by the unpaired t-test. *p < 0.05. All figures were generated from original experimental data by the authors using Prism 7 (GraphPad Software, GraphPad, San Diego, USA). mRNA: messenger RNA; siRNA: small interfering RNA

The hepatic mRNA expression level was significantly reduced by 85% in the *Elovl3*-specific siRNA-injected group compared to that in the negative siRNA-injected group 48 h after injection (Figure1).

**Figure 2:**
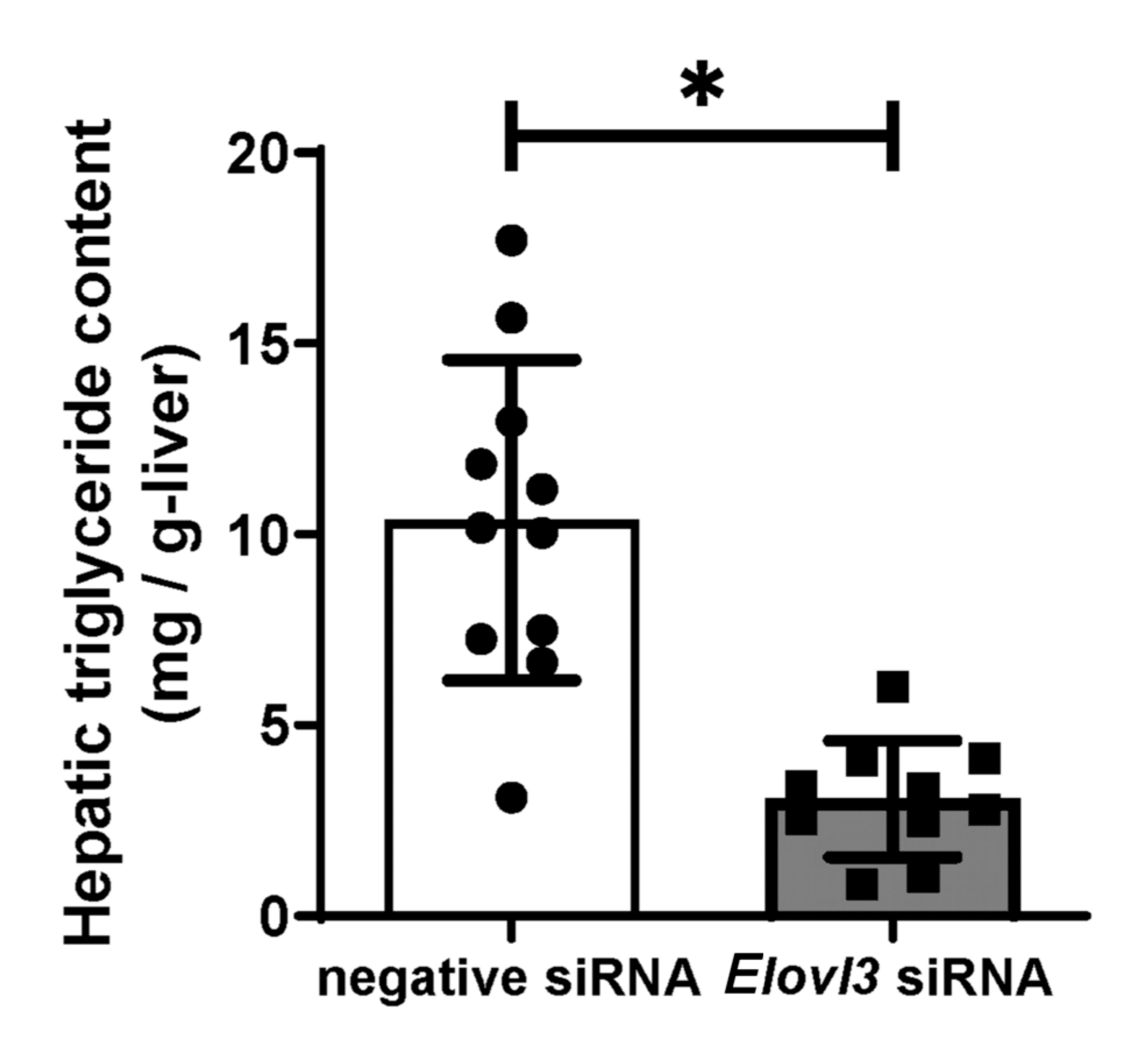
Hepatic triglyceride contents in the Elovl3 siRNA group (n=7) compared to those in the negative siRNA group (n=11). Data values correspond to mean values, and error bars represent the standard deviation of the means. Differences among groups were assessed by the unpaired t-test. *p < 0.05. siRNA: small interfering RNA

To evaluate the effect of reduced hepatic mRNA expression of *Elovl3* on lipid accumulation, the hepatic triglyceride content was measured. As shown in Figure 2, the hepatic triglyceride content in the *Elovl3* siRNA group was significantly decreased after a single 6-hour sleep deprivation (10.4±4.2 vs 3.1±1.5 mg/g-tissue, negative siRNA vs *Elovl3* siRNA, respectively, p < 0.01).

**Figure 3:**
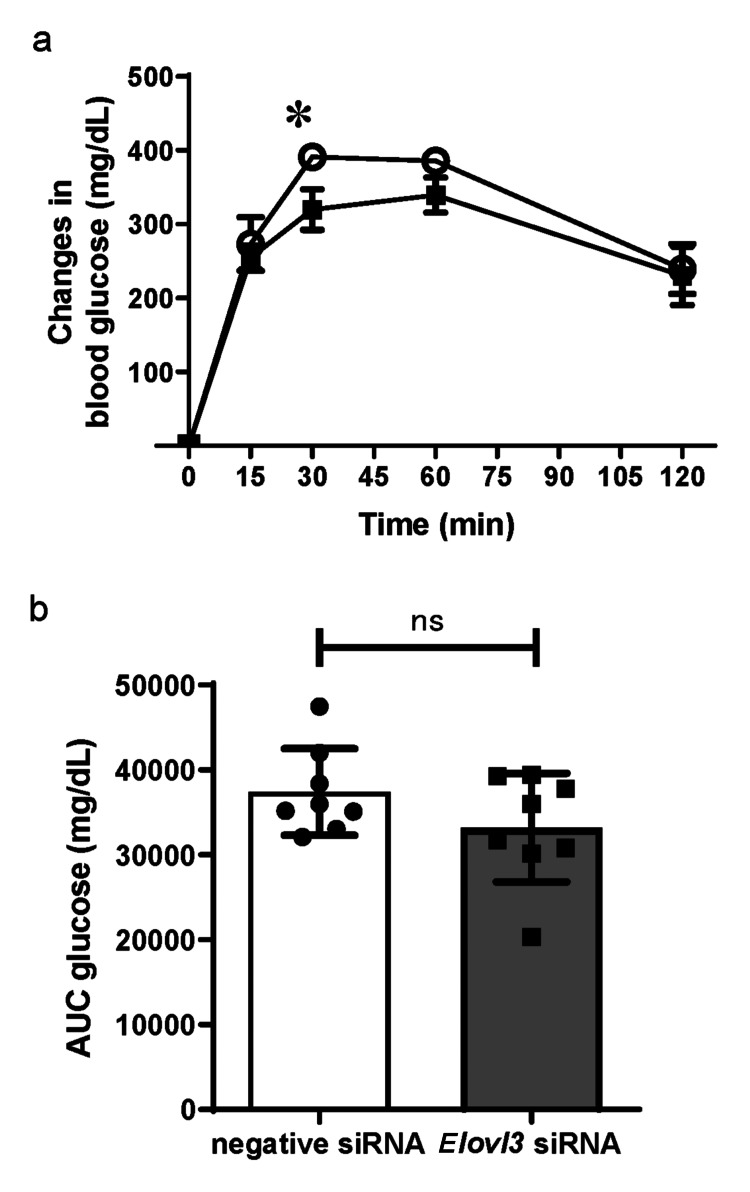
Intraperitoneal glucose tolerance test. (a) Changes in blood glucose concentrations after intraperitoneal glucose injection (2.0 g/kg-BW) in the *Elovl3 *siRNA (black square, n=8) and negative siRNA (white circle, n=8) groups after sleep deprivation induced by the gentle handling method over a single 6-hour session. (b) The area under the curve (AUC) of glucose was calculated for individual mice and averaged for the two groups. Data values correspond to mean values, whereas error bars represent the standard deviation of the means. Differences among groups were assessed using two-way analysis of variance followed by Tukey’s post hoc test (a) or unpaired t-test (b). *p < 0.05. ns; not significant. siRNA: small interfering RNA

To assess the effects of reduced hepatic triglyceride levels on glucose metabolism, we performed an ipGTT after the sleep deprivation period. The ipGTT (Figure 3a) revealed that the increase in blood glucose concentrations at 30 min from baseline was significantly smaller in the *Elovl3* siRNA group than in the negative siRNA group (319.6±78.0 vs 391.0±31.7 mg/dL, respectively, p < 0.05) while baseline glucose concentrations at time 0 were comparable between the groups (155.1±27.5 vs 141.1±37.6 mg/dL, respectively). However, no significant differences were found in the overall glucose levels between the treatments, as calculated by the area under the curve (Figure 3b).

**Figure 4:**
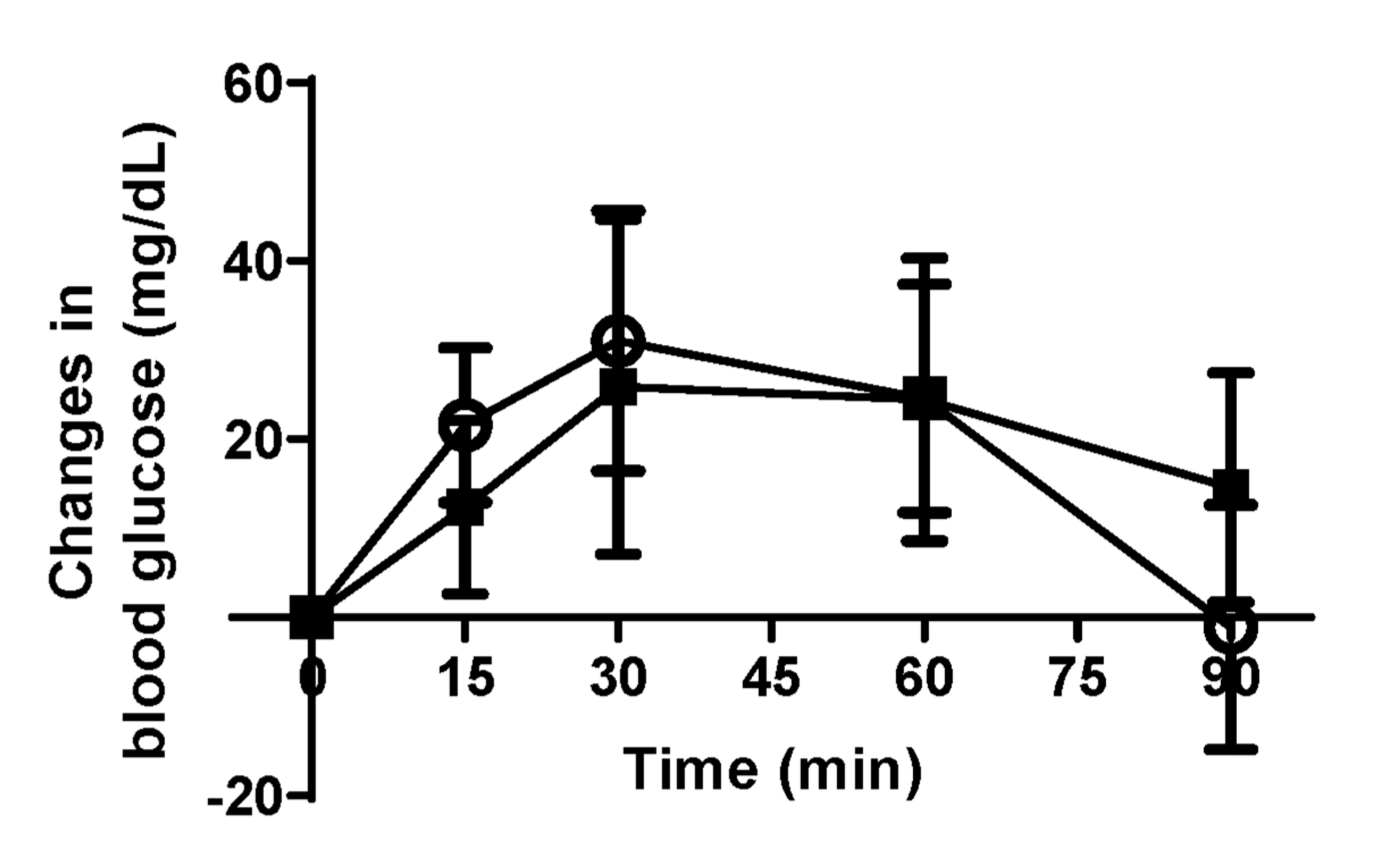
Pyruvate challenge test. Changes in blood glucose concentrations in the *Elovl3* siRNA (black square) and negative siRNA (white circle) groups measured following intraperitoneal pyruvate injection (1.5 g/kg) after 6-hour sleep deprivation. n=9 per group. Data points correspond to mean values, whereas error bars represent the standard deviation of the means. siRNA: small interfering RNA

**Figure 5:**
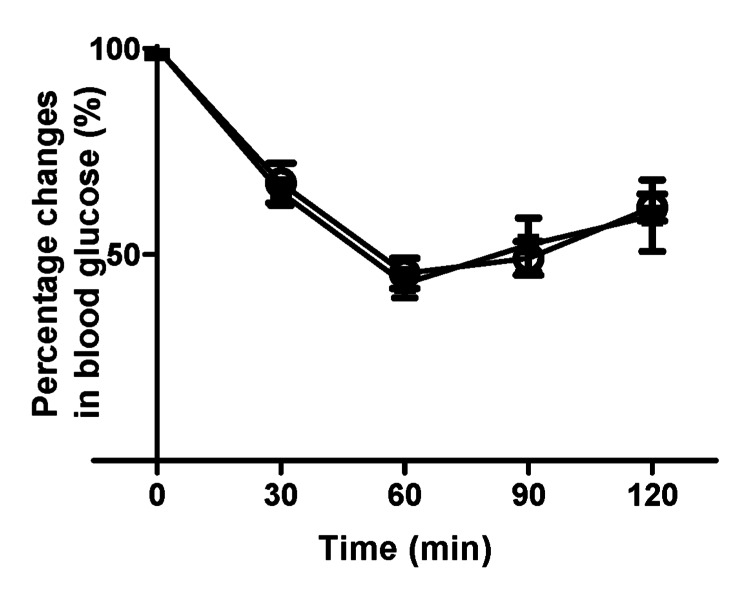
Insulin tolerance test. Percentage changes in blood glucose concentrations in the *Elovl3* siRNA (black square) and negative siRNA (white circle) groups following intraperitoneal insulin injection (0.75 U/kg) measured after 6-hour sleep deprivation. n=10 per group. Data points correspond to mean values, and error bars represent the standard deviation of the means. siRNA: small interfering RNA

To assess hepatic insulin sensitivity, we performed further investigations using the pyruvate challenge and insulin tolerance tests. No significant differences were observed between the *Elovl3* siRNA and negative siRNA groups in either the pyruvate challenge or insulin tolerance test (Figures 4, 5).

**Figure 6:**
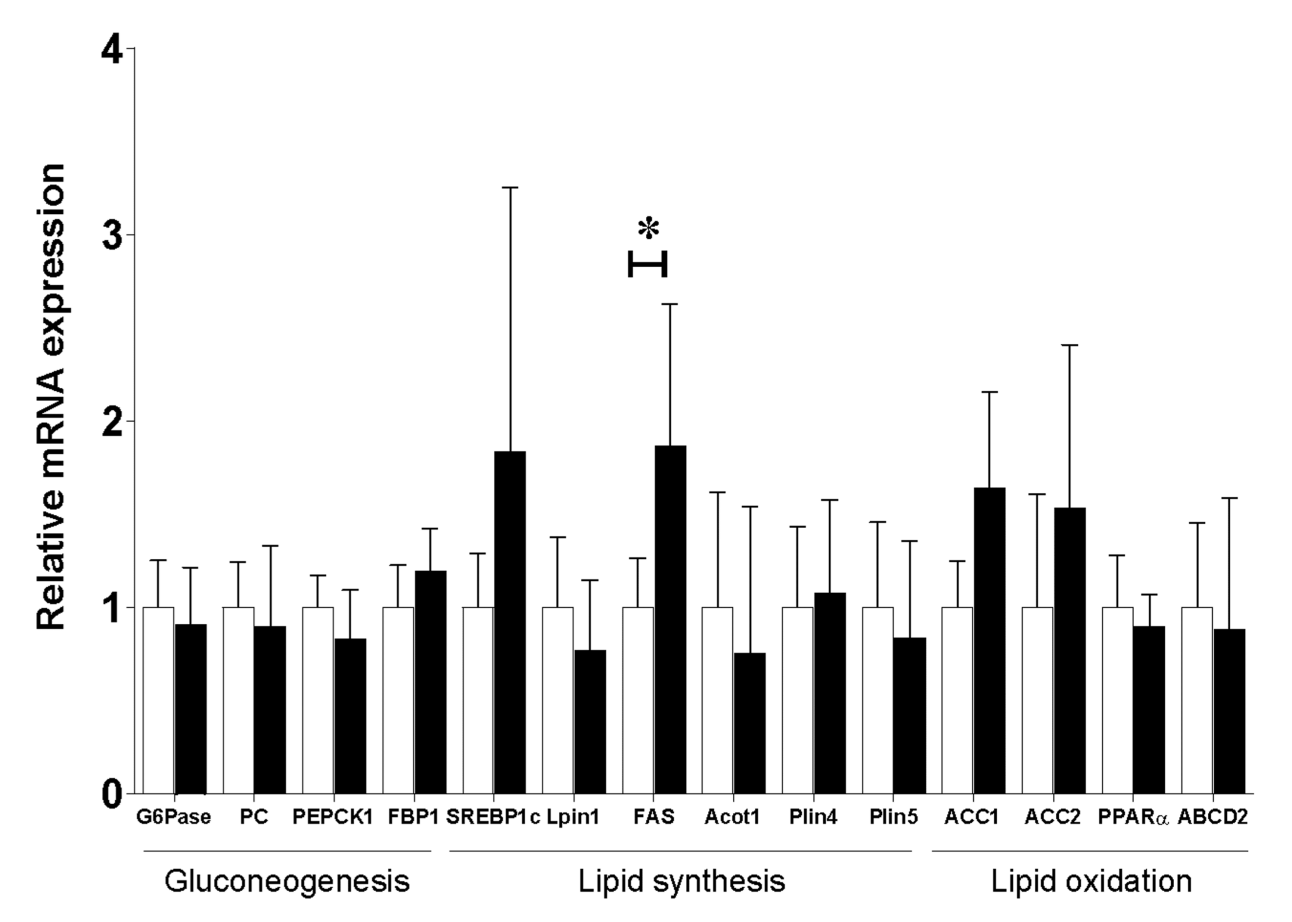
Comparison of mRNA expression levels of hepatic gluconeogenesis, lipogenesis, and lipid oxidation-related genes between the Elovl3 siRNA-and negative siRNA-treated groups. The black bars represent the *Elovl3* siRNA group (n=7), and the white bars represent the negative siRNA group (n=11). The average mRNA expression level of the negative siRNA group was set as 1. Data are presented as mean ± standard deviation. Differences among groups were assessed by the unpaired t-test. *p < 0.05. G6Pase: glucose-6-phosphatase; PC: pyruvate carboxylase; PEPCK1: phosphoenolpyruvate carboxykinase 1; FBP1: fructose-1,6-bisphosphatase; SREBP1c: sterol regulatory element binding transcription factor 1c; *Lpin1*: lipin 1; FAS: fatty acid synthase; *Acot1*: acyl-CoA thioesterase 1; *Plin4*: perilipin 4; *Plin5*: perilipin 5; ACC1: acetyl-coenzyme A carboxylase 1; ACC2: acetyl-coenzyme A carboxylase; PPARα: peroxisome proliferator-activated receptor α; ABCD2: ATP-binding cassette sub family D member 2.

Furthermore, quantitative RT-PCR was used to assess the hepatic genes known to be involved in promoting gluconeogenesis, lipid synthesis, and lipid oxidation. No significant differences were observed between the treatments regarding the genes that promote gluconeogenesis. The gene expression of fatty acid synthase was significantly higher in the *Elovl3* siRNA group than in the negative siRNA group. However, other genes known to be involved in promoting lipogenesis, such as Lipin 1 (*Lpin1*), Acyl-CoA thioesterase 1 (*Acot1*), and Perilipin 4 and 5 (Plin4, Plin5), exhibited similar expression levels between the two groups. Regarding the genes that promote lipid oxidation, no significant differences were found in acetyl-CoA carboxylases 1 and 2 and peroxisome proliferator-activated receptor α between the treatments (Figure *6*).

## Discussion

In this study, hepatic lipid accumulation was significantly reduced by preventing sleep deprivation-induced upregulation of hepatic *Elovl3* expression. Although the blood glucose levels at 30 min during ipGTT were significantly lower in the *Elovl3* siRNA group, no significant difference was observed in the area under the curve of glucose, indicating that the overall glucose response was not significantly improved.

*Elovl3* was identified as a lipogenesis-related gene involved in the synthesis of C20-C24 saturated and monounsaturated very long-chain fatty acids [21,22]. Previous studies have provided evidence that *Elovl3* expression exhibits robust circadian rhythmicity in the mouse liver [21,25-27]. Additionally, we previously reported that *Elovl3* mRNA expression increased 2.7-fold after a single 6-hour sleep deprivation [20]. In this study, compared to the negative siRNA group, the expression of *Elovl3* was significantly reduced in the *Elovl3* siRNA group (Figure *1*, p < 0.01), indicating that the increase in *Elovl3* expression induced by sleep deprivation was completely negated 48 h after the injection. In addition, our previous study indicated that a single 6-hour sleep deprivation induced an increase in hepatic triglyceride content by 67.9% compared to that in the control group in C57BL/6J mice [20]; however, injection of *Elovl3* siRNA succeeded in reducing it by 70.4%. These results suggest that injection with *Elovl3* siRNA can ameliorate the increased hepatic mRNA levels and improve hepatic lipid accumulation caused by sleep deprivation.

Interestingly, we observed a significant increase in fatty acid synthase gene expression in the *Elovl3* siRNA group, despite the overall reduction in hepatic triglyceride levels. Although *Elovl3* directly affects very-long-chain fatty acid synthesis, the compensatory upregulation of fatty acid synthase, a key enzyme in *de novo* lipogenesis, suggests potential metabolic reprogramming or feedback mechanisms. This unexpected finding may contribute to the limited overall improvement in glucose metabolism, at least in part, and warrants further investigation into the interplay between *Elovl3* suppression and other lipogenic pathways. Specifically, because protein-level expression, enzyme activity, and lipid flux were not examined, this finding should be interpreted cautiously as a possible compensatory transcriptional response rather than as evidence of a confirmed functional mechanism.

Insulin sensitivity assessed using the insulin tolerance test revealed no differences between the groups (Figure 4). The pathogenesis of T2DM is associated with hepatic and muscle insulin resistance caused by ectopic lipid accumulation in the liver and skeletal muscle [28,29]. A previous report revealed that reducing the hepatic lipid content leads to an improvement in hepatic insulin resistance [30]. In this study, although the reduction in hepatic triglyceride content led to a significant reduction in glucose elevation at 30 min in the ipGTT in the *Elovl3* siRNA group compared to the negative siRNA group (p < 0.05), no significant difference was observed in hepatic gluconeogenesis assessed by the pyruvate challenge test and hepatic gene expression that promotes gluconeogenesis. Interestingly, some previous studies have suggested no correlation between hepatic triglyceride content and hepatic glucose production [31,32].

In contrast, hepatic diacylglycerol (DAG) content has been reported to be the best predictor of insulin resistance and is responsible for 64% of the variability in insulin sensitivity [33]. In this study, we did not perform hepatic intracellular lipid fractionation because in the previous study [20], we had evaluated hepatic DAGs composed of C16:1-C16:1, C18:0-C18:0, and C18:1-C18:1; however, no significant differences in the hepatic DAG content were found between the sleep deprivation and control groups. Recently, sn-1,2-DAG in the hepatic intracellular lipid droplet versus plasma membrane has been considered to be associated with hepatic protein kinase C epsilon (PKCε) activation, leading to hepatic insulin resistance, in contrast to DAGs in other compartments or 1,3- or 2,3- DAG stereoisomers [34]. Thus, the content of sn-1,2-DAG in the hepatic intracellular lipid droplet could be attributed to the pathogenesis of insulin resistance-induced glucose intolerance; however, the contribution of the reduction in hepatic triglyceride content to hepatic glucose production should be small.

Despite the important findings of our study that suppression of *Elovl3* expression can ameliorate sleep deprivation-induced hepatic steatosis, our study has several limitations. First, as it is a model for assessing the acute implications of sleep deprivation, a chronic sleep deprivation model should be developed to further assess changes in these parameters and validate our findings in the long term. Second, our study focused on the effect of suppressing hepatic *Elovl3* expression by considering *Elovl3* as a treatment target; however, the overall implications of suppressing *Elovl3* expression at the whole-body level were not studied. Third, the gentle-handling protocol and temporary movement restriction may have introduced stress-related neuroendocrine effects. Although this approach avoids forced locomotion and has been widely used to minimize excessive experimental stress, we cannot completely exclude the possibility that stress contributed, at least in part, to the observed metabolic changes. Finally, this study was conducted in a rodent model system, and further studies are needed to verify whether suppressing *Elovl3* expression applies to humans.

## Conclusions

In conclusion, our results suggest that suppression of *Elovl3* expression could ameliorate sleep deprivation-induced hepatic steatosis, as evidenced by a 2.7-fold increase in hepatic *Elovl3* expression after sleep deprivation, an approximately 85% reduction in *Elovl3* mRNA levels following siRNA administration, and a significant reduction in hepatic triglyceride content (10.4±4.2 vs 3.1±1.5 mg/g tissue, p < 0.01); however, its effects on glucose metabolism are limited. *Elovl3* may be a potential therapeutic target for sleep deprivation-induced hepatic steatosis, although additional interventions are necessary to treat glucose intolerance. Our study provides insights and a basis for future studies aimed at treating sleep deprivation-induced hepatic steatosis in humans.

## References

[1] Lim DC, Najafi A, Afifi L (2023). The need to promote sleep health in public health agendas across the globe. Lancet Public Health.

[2] Benjafield AV, Sert Kuniyoshi FH, Malhotra A (2025). Estimation of the global prevalence and burden of insomnia: a systematic literature review-based analysis. Sleep Med Rev.

[3] Koffel E, Ancoli-Israel S, Zee P, Dzierzewski JM (2023). Sleep health and aging: Recommendations for promoting healthy sleep among older adults: a National Sleep Foundation report. Sleep Health.

[4] Pappas JA, Miner B (2024). Sleep deficiency in the elderly. Sleep Med Clin.

[5] Zheng NS, Annis J, Master H (2024). Sleep patterns and risk of chronic disease as measured by long-term monitoring with commercial wearable devices in the All of Us Research Program. Nat Med.

[6] Darraj A (2023). The link between sleeping and type 2 diabetes: a systematic review. Cureus.

[7] Wright AK, Huang T, Carr MJ (2025). Clinical utility of self-reported sleep duration and insomnia symptoms in type 2 diabetes prediction. Diabetologia.

[8] Zhang F, Xue Y, Li W (2025). Exploring the impact of sleep duration and sleep disorders on metabolic dysfunction-associated steatotic liver disease in older adults. BMC Geriatr.

[9] Yang J, Zhang K, Xi Z, Ma Y, Shao C, Wang W, Tang YD (2023). Short sleep duration and the risk of nonalcoholic fatty liver disease/metabolic associated fatty liver disease: a systematic review and meta-analysis. Sleep Breath.

[10] Spiegel K, Tasali E, Leproult R, Van Cauter E (2009). Effects of poor and short sleep on glucose metabolism and obesity risk. Nat Rev Endocrinol.

[11] Barf RP, Meerlo P, Scheurink AJ (2010). Chronic sleep disturbance impairs glucose homeostasis in rats. Int J Endocrinol.

[12] Rechtschaffen A, Bergmann BM (2002). Sleep deprivation in the rat: an update of the 1989 paper. Sleep.

[13] Everson CA, Szabo A (2009). Recurrent restriction of sleep and inadequate recuperation induce both adaptive changes and pathological outcomes. Am J Physiol Regul Integr Comp Physiol.

[14] Penev PD (2012). Update on energy homeostasis and insufficient sleep. J Clin Endocrinol Metab.

[15] Damiola F, Le Minh N, Preitner N, Kornmann B, Fleury-Olela F, Schibler U (2000). Restricted feeding uncouples circadian oscillators in peripheral tissues from the central pacemaker in the suprachiasmatic nucleus. Genes Dev.

[16] Tahara Y, Shibata S (2016). Circadian rhythms of liver physiology and disease: experimental and clinical evidence. Nat Rev Gastroenterol Hepatol.

[17] Speksnijder EM, Bisschop PH, Siegelaar SE, Stenvers DJ, Kalsbeek A (2024). Circadian desynchrony and glucose metabolism. J Pineal Res.

[18] Panasiuk A, Tarasewicz M, Chodowiec A, Łokić A, Gan K (2024). Biological rhythms of the liver. Clin Exp Hepatol.

[19] Verdelho Machado M (2024). Circadian deregulation: back facing the sun toward metabolic dysfunction-associated steatotic liver disease (MASLD) development. Nutrients.

[20] Shigiyama F, Kumashiro N, Tsuneoka Y (2018). Mechanisms of sleep deprivation-induced hepatic steatosis and insulin resistance in mice. Am J Physiol Endocrinol Metab.

[21] Chen H, Gao L, Yang D (2019). Coordination between the circadian clock and androgen signaling is required to sustain rhythmic expression of Elovl3 in mouse liver. J Biol Chem.

[22] Westerberg R, Tvrdik P, Undén AB (2004). Role for ELOVL3 and fatty acid chain length in development of hair and skin function. J Biol Chem.

[23] Kumashiro N, Tamura Y, Uchida T (2008). Impact of oxidative stress and peroxisome proliferator-activated receptor gamma coactivator-1alpha in hepatic insulin resistance. Diabetes.

[24] Kumashiro N, Beddow SA, Vatner DF (2013). Targeting pyruvate carboxylase reduces gluconeogenesis and adiposity and improves insulin resistance. Diabetes.

[25] Brolinson A, Fourcade S, Jakobsson A, Pujol A, Jacobsson A (2008). Steroid hormones control circadian Elovl3 expression in mouse liver. Endocrinology.

[26] Anzulovich A, Mir A, Brewer M, Ferreyra G, Vinson C, Baler R (2006). Elovl3: a model gene to dissect homeostatic links between the circadian clock and nutritional status. J Lipid Res.

[27] Zadravec D, Brolinson A, Fisher RM (2010). Ablation of the very-long-chain fatty acid elongase ELOVL3 in mice leads to constrained lipid storage and resistance to diet-induced obesity. FASEB J.

[28] Samuel VT, Shulman GI (2016). The pathogenesis of insulin resistance: integrating signaling pathways and substrate flux. J Clin Invest.

[29] Shulman GI (2000). Cellular mechanisms of insulin resistance. J Clin Invest.

[30] Petersen KF, Dufour S, Befroy D, Lehrke M, Hendler RE, Shulman GI (2005). Reversal of nonalcoholic hepatic steatosis, hepatic insulin resistance, and hyperglycemia by moderate weight reduction in patients with type 2 diabetes. Diabetes.

[31] Vatner DF, Weismann D, Beddow SA (2013). Thyroid hormone receptor-β agonists prevent hepatic steatosis in fat-fed rats but impair insulin sensitivity via discrete pathways. Am J Physiol Endocrinol Metab.

[32] Cantley JL, Yoshimura T, Camporez JP (2013). CGI-58 knockdown sequesters diacylglycerols in lipid droplets/ER-preventing diacylglycerol-mediated hepatic insulin resistance. Proc Natl Acad Sci U S A.

[33] Kumashiro N, Erion DM, Zhang D (2011). Cellular mechanism of insulin resistance in nonalcoholic fatty liver disease. Proc Natl Acad Sci U S A.

[34] Abulizi A, Vatner DF, Ye Z (2020). Membrane-bound sn-1,2-diacylglycerols explain the dissociation of hepatic insulin resistance from hepatic steatosis in MTTP knockout mice. J Lipid Res.

